# A non-trauma-focused equine-assisted intervention was associated with reductions in all four PTSD symptom clusters in treatment-resistant military veterans

**DOI:** 10.3389/fpsyg.2026.1787669

**Published:** 2026-06-03

**Authors:** C. M. Kapteijn, R. A. Fennema, R. van Huffelen, N. Endenburg, E. Vermetten, T. B. Rodenburg

**Affiliations:** 1 Animals in Science and Society, Department of Population Health Sciences, Faculty of Veterinary Medicine, Utrecht University, Utrecht, Netherlands; 2 GGz Centraal, Amersfoort, Netherlands; 3 Mentrum Psychiatric Hospital, Amsterdam, Netherlands; 4 Department of Psychiatry, Leiden University Medical Centre, Leiden, Netherlands

**Keywords:** clinically significant change (CSC), equine-assisted intervention, PCL-5, post-traumatic stress disorder (PSTD), reliable change index (RCI), symptom clusters, military veterans

## Abstract

**Introduction:**

Veterans are at greater risk of developing post-traumatic stress disorder (PTSD) and show a poorer response to first-line treatments than civilians. Although equine-assisted interventions (EAIs) represent a promising alternative for military veterans,the underlying mechanisms remain uncertain, and their effectiveness across different PTSD symptom clusters has yet to be established. This study examined the effects of a non-trauma-focused EAI on specific PTSD symptoms in treatment-resistant military veterans.

**Material and methods:**

Participants in the intervention were military veterans diagnosed with PTSD by their psychological or psychiatric referrer, all of whom had received previous first-line trauma treatment prior to study enrolment. The dataset included 43 veterans who completed the Dutch PTSD Checklist (PCL) for the Diagnostic and Statistical Manual of Mental Disorders-5 (DSM-5) at week 1 and week 24, and who met the PTSD criteria at baseline according to the PCL-5. The EAI was a 12-week group program during which veterans had individual interactions with a horse once a week from 10:00 to 15:00. PTSD symptoms were assessed using the PCL-5 at weeks 1, 4, 8, 12, 24, and 52.

**Results:**

A linear mixed model showed that scores for all four PTSD symptom clusters significantly improved over the course of the program. Cluster B (intrusion) and cluster C (avoidance) decreased even further after termination of the program at week 12, demonstrating additional long-term effects. Cluster D (negative changes in cognition and mood) and Cluster E (arousal and reactivity) remained low but did not decrease further after week 12. In addition, the reliable change index (RCI) and clinically significant change (CSC) were calculated, resulting in suboptimal (51.2%; *n* = 22), improved (23.3%; *n* = 10), and recovered (25.6%; *n* = 11) treatment-outcome groups.

**Discussion:**

This indicates that 25.6% achieved both reliable and clinically meaning full symptom reduction in response to the program, and 23.3% demonstrated statistically reliable but not fully clinically significant improvement, while 51.2% did not show any improvement. Although responsiveness over time differed, all PTSD symptom clusters improved significantly during the EAI program in treatment-resistant military veterans. Suggesting that further research with controlled study designs on the effectiveness of EAI as an adjunctive intervention to TFT for military veterans with residuals symptoms would be of value.

## 1 Introduction

Post-traumatic stress disorder (PTSD) is a mental health disorder that occurs after exposure to one or more traumatic events and is characterized by symptoms across four clusters: intrusion symptoms, persistent avoidance of stimuli, negative alterations in cognition and mood, and alterations in arousal and reactivity associated with the traumatic event(s) (American Psychiatric Association, 2013). Existing treatment guidelines for PTSD recommend trauma-focused therapies (TFTs), such as cognitive behavioral therapy (Lely et al., 2019), prolonged exposure (Rauch et al., 2012), and eye movement desensitization and reprocessing (Korn, 2009), as first-line interventions, with pharmacotherapy as an adjunct when needed (Charney et al., 2018; Forbes et al., 2010; Melani et al., 2024; Steenkamp et al., 2015; Watkins et al., 2018). Despite the availability of several evidence-based treatments, a substantial proportion of patients continue to experience clinically significant PTSD symptoms after first-line treatment (Levi et al., 2021; Semmlinger et al., 2024; Wesseling et al., 2025). A recent review reported an average non-response rate of 39.23% across 86 studies evaluating PTSD treatments (Semmlinger et al., 2024).

The prevalence of PTSD is relatively high among military veterans, with rates reported of up to 34.84% (Gates et al., 2012; Hoge et al., 2004; van der Wal et al., 2020; Xue et al., 2015). Moreover, military veterans tend to benefit less from first-line treatments compared with other populations (Coventry et al., 2020). Approximately two-thirds of military veterans continue to meet diagnostic criteria for PTSD following first-line treatment (Levi et al., 2021; Steenkamp et al., 2015; Steenkamp et al., 2020). This highlights the need to investigate additional treatment options for military veterans with PTSD who do not respond adequately to first-line interventions (Steenkamp et al., 2015), henceforth referred to in this study as treatment-resistant PTSD. Although TFTs are generally considered more effective than non-trauma-focused therapies (non-TFTs) that primarily target current functioning (Bisson et al., 2007; Grech and Grech, 2020), other evidence suggests that non-TFTs may also provide meaningful benefits (Bisson et al., 2013; Steenkamp et al., 2020). Furthermore, drop-out rates appear to be lower for non-TFTs than for TFTs (Edwards-Stewart et al., 2021). A potential complementary approach is equine-assisted interventions (EAIs). EAIs have been proposed as a promising non-trauma-focused addition for military veterans with PTSD. However, the existing evidence regarding the effectiveness of EAIs is limited and is characterized by low methodological quality (Cornelius-White et al., 2024; Kapteijn et al., 2025a; Marchand, 2023; Provan et al., 2024). In addition, the mechanisms of action through which EAI may influence PTSD symptoms are not well understood and require further investigation (Beetz, 2017; Kapteijn et al., 2025a; Marchand, 2023).

Examining symptom clusters may provide further insight into the mechanisms through which EAIs exert their effects and may support more personalized treatment approaches by focusing on individual symptom patterns (Grasser and Javanbakht, 2019). The four symptom clusters within the PTSD Checklist for the DSM-5 (PCL-5) reflect distinct dimensions of PTSD symptomatology (American Psychiatric Association, 2013; Friedman, 2013; Melani et al., 2024). However, the majority of research examining treatment outcomes in PTSD focuses on overall symptom severity by reporting total PCL-5 scores, rather than changes in specific symptom clusters (Grasser and Javanbakht, 2019; Larsen et al., 2019). This is also the case in most studies investigating the effects of EAIs in PTSD (Arnon et al., 2020; Burton et al., 2019; Earles et al., 2015; Fisher et al., 2021; Johnson et al., 2018; Lanning et al., 2017; Lanning et al., 2018; Monroe et al., 2019; Nevins et al., 2013; Romaniuk et al., 2018; Wharton et al., 2019; Willmund et al., 2021; Zhu et al., 2021). To date, only one study specifically examined the effects of EAIs on individual PTSD symptom clusters, reporting significant reductions primarily in cluster E (arousal and reactivity) (Malinowski et al., 2018). Investigating symptom clusters may provide additional insight into which aspects of PTSD are most responsive to EAIs.

In addition to examining symptom clusters, research has emphasized the importance of moving beyond population-level effect sizes toward evaluating clinical significance at the individual level (Jacobson and Truax, 1991). Examining residual symptoms in individuals may increase understanding of treatment effects and improve treatment efficacy (Larsen et al., 2019). Significant changes in symptom scores do not necessarily indicate that the change is clinically meaningful for an individual patient (Marx et al., 2022). The reliable change index (RCI) may be used to determine whether the observed changes exceed measurement error and therefore represent reliable improvement or deterioration (Blampied, 2022). When combined with criteria for clinically significant change (CSC), this approach allows researchers to evaluate whether symptom changes correspond to meaningful clinical improvement. Therefore, this study examines changes in PTSD symptom clusters following EAI and evaluates clinical significance using the RCI and CSC at both the symptom and cluster level.

## 2 Method

### 2.1 Participants

In total, 72 military veterans participated in this study from 2020 to 2025. The sample was self-selected, and inclusion criteria were a former PTSD diagnosis confirmed by a psychological or psychiatric referrer and previous receipt of first-line treatment. Exclusion criteria for participation in the program included current addiction, ongoing therapy during participation, involvement in legal claims against the Ministry of Defence, and/or aggressive behavior towards people or animals. Data of 43 veterans (33 men, 10 women; average age of 46.9 ± SD 9.9 years), who continued to meet PTSD diagnostic criteria at admission despite previous first-line treatment and completed the PCL-5 at week 1 and week 24, were included in the dataset. In total, 29 participants were excluded from the dataset because they did not meet the PTSD diagnostic criteria based on the PCL-5 score in week 1 and/or did not complete the PCL-5 at week 1 or week 24. A total of 6 veterans did not finalize the program, resulting in a dropout rate of 8.3%. PTSD diagnosis was defined as a score ≥2 on at least one symptom in cluster B (intrusion), one symptom in cluster C (avoidance), two symptoms in cluster D (negative changes in mood or cognition), and two symptoms in cluster E (arousal and reactivity) on the PCL-5 (American Psychiatric Association, 2013).

### 2.2 Intervention

The EAI program consisted of 12 non-trauma-focused group sessions conducted once a week and one follow-up session after a three-month interval (week 24), all lasting from 10:00 to 15:00. Interactions were performed individually, with veterans taking turns performing the activities. Horses were placed in an octagon-shaped ring (11.4 m diameter), demarcated with temporary fencing poles and white ribbons, within an indoor riding arena (20 m × 40 m). Each day consisted of instructions (45 min), a free activity (*F*: 22.2 ± 2.8 min), a directed activity (D: 30.2 ± 6.3 min), and a group evaluation/reflection (45 min). Exceptions were week 1, which had no directed activity, and week 24, which had no free activity. During the free activity (*F*), veterans focused mainly on making physical contact with the horse and relaxation. From 12:30 to 13:30, veterans had a lunch break. In the afternoon, veterans performed a specific assignment together with the horse (D), including both unmounted and mounted activities such as grooming, groundwork, and riding. The intervention was delivered by a mental healthcare psychologist and a behavioral biologist specialized in horse behavior, under the guidance of a psychiatrist and psychologist specialized in PTSD in military veterans. This core team of professionals was present across all groups to deliver the intervention according to a standardized protocol. For further details, see Table 1.

**Table 1:** The 12-week equine-assisted intervention program showing when free and directed interactions were performed, including a short description of the activities, their location, and when the PCL-5 questionnaire was administered.

Week	Part of the day	Interaction type	Description	Location	Psychometric questionnaire
1	Morning		–		PCL-5
	Afternoon	Free	Physical contact with the horse and relaxation	Ring	
2	Morning	Free	Physical contact with the horse and relaxation	Ring	
	Afternoon	Directed	Grooming and hoof picking	Ring	
3	Morning	Free	Physical contact with the horse and relaxation	Ring	
	Afternoon	Directed	Haltering and leading the horse	Ring	
4	Morning	Free	Physical contact with the horse and relaxation	Ring	PCL-5
	Afternoon	Directed	Lunging the horse (including trotting)	Ring	
5	Morning	Free	Physical contact with the horse and relaxation	Ring	
	Afternoon	Directed	Groundwork; leading the horse around traffic cones in a figure eight	Ring	
6	Morning	Free	Physical contact with the horse and relaxation	Ring	
	Afternoon	Directed	Groundwork; leading the horse to step over 3 caveletti beams	Ring	
7	Morning	Free	Physical contact with the horse and relaxation	Ring	
	Afternoon	Directed	Groundwork; leading the horse to walk over a plastic cover	Ring	
8	Morning	Free	Physical contact with the horse and relaxation	Ring	PCL-5
	Afternoon	Directed	Groundwork; leading the horse through an obstacle course	Riding arena	
9	Morning	Free	Physical contact with the horse and relaxation	Ring	
	Afternoon	Directed	Riding on a bareback pad while on a lead rope with a handler	Ring	
10	Morning	Free	Physical contact with the horse and relaxation	Ring	
	Afternoon	Directed	Riding with a saddle and bridle while on a lunge line	Ring	
11	Morning	Free	Physical contact with the horse and relaxation	Ring	
	Afternoon	Directed	Riding with a saddle and bridle loose within an individual ring	Ring	
12	Morning	Free	Physical contact with the horse and relaxation	Ring	PCL-5
	Afternoon	Directed	Riding with a saddle and bridle while in a group in the riding arena	Riding arena	
24	Morning		–		PCL-5
	Afternoon	Directed	Riding with a saddle and bridle while in a group in the riding arena	Riding arena	
52		–	–	At home	PCL-5

### 2.3 Horses

Six horses of an unspecified breed from a Dutch riding school (Stal Groenendaal, Bunschoten, the Netherlands) were involved in the interventions; one mare and five geldings with an average wither height of 163.8 ± 8.0 cm and an average age of 20.2 ± 6.1 years. All horses were clinically healthy and assessed for soundness, and their welfare was monitored throughout the program by measuring behavioral and physiological parameters (Kapteijn et al., 2025b). The horses were selected based on their stable temperament and prior experience in riding lessons for mentally and physically disabled people; none had prior experience with EAIs or exposure to veterans with PTSD. The horses were housed in individual stalls with straw bedding, *ad libitum* hay (distributed at 8:00 and 16:00), and concentrate feed twice daily (12:00 and 19:30). Daily turnout occurred in a group paddock 1.5–2 h daily. In addition to their routine, horses were ridden for 1–3 h per day alongside participation in the EAI sessions. All baseline and intervention procedures were performed in an individual octagon-shaped ring (11.4 m diameter) with all horses within visual contact of each other, but unable to contact each other physically. Horses were under continuous supervision by the research team and were able to move freely in the ring or be restrained with a lead rope when necessary.

### 2.4 PCL-5

Participants completed the PTSD Checklist for the DSM-5 (PCL-5) at the beginning of the intervention, in weeks 4, 8, and 12, at the 3-month follow-up (week 24), and at the 1-year follow-up (week 52). The number of veterans meeting the criteria for a PTSD diagnosis was calculated at week 1 and week 24. The PCL-5 is a 20-item self-rating Likert-scale questionnaire that assesses the DSM-5 symptoms of PTSD, divided into four symptom clusters. The PCL-5 has excellent psychometric properties in veteran samples (Bovin et al., 2016; Hoeboer et al., 2024). The Dutch version was used in this study, as it has been validated for Dutch trauma-exposed adults and showed excellent internal consistency, reliability, and high criterion validity (Van Praag et al., 2020). A factor analysis showed a good fit for the most frequently tested four-factor DSM-5 model (Van Praag et al., 2020).

### 2.5 Statistical analysis

To determine significant changes in cluster scores over time, a linear mixed model was performed using SPSS Statistics (version 30.0.0, IBM Corp.) for all four clusters (B, C, D, and E of the PCL-5). The covariance structure was selected based on the Akaike information criterion (AIC) for each cluster separately, with lower values indicating a better fit (Chan, 2004; Duricki et al., 2016). In the results section, the selected covariance structure is reported for each outcome measure. For all clusters, participants were included as a random factor with the intercept included, week was treated as the repeated factor, number of deployments was included as a covariate, and gender, existing medication use, experience with horses, and group were specified as fixed factors. Interaction effects between week and the other factors were also included, with the exception of the interaction between week and group, as this negatively affected convergence due to the large number of levels. Model selection was conducted through backward deletion (Chan, 2004; Duricki et al., 2016), based on a reduction of the AIC of more than 2 points (Burnham and Anderson, 2004). Residuals for all clusters were normally distributed according to the exact Kolmogorov–Smirnov one-sample test.

In addition, differences between veterans who appeared to benefit from the treatment and those who did not were further investigated at both the cluster and individual symptom level. Calculating the RCI and the CSC allows the investigation of subgroups of treatment responders (Miles et al., 2023). A participant was considered to have experienced a statistically reliable change when their RCI score exceeded 1.96 (*p* < 0.05), indicating that the likelihood of the change being due to random variation or measurement error was <5% (Jacobson and Truax, 1991; Marx et al., 2022; Miles et al., 2023). To meet the RCI criteria, participants required post-intervention scores at least 12 points lower than their admission scores. The CSC was used to assess whether the change was clinically relevant and whether a respondent was more likely to belong to the non-disordered population than to the disordered population (Jacobson and Truax, 1991; Marx et al., 2022). Participants were required to have post-intervention scores at least two standard deviations below the mean of all randomized individuals at admission to meet the CSC criteria (Jacobson and Truax, 1991). Consequently, this established a CSC cut-off score of 31. For each symptom, the decrease was calculated as the absolute difference between two percentages in percentage points (pp). The RCI and the CSC scores were calculated using Microsoft Excel for Mac v16.98. Individuals were classified into three response categories: recovered individuals (surpassing both CSC and RCI cut-offs in the positive direction), improved individuals (surpassing the RCI but not the CSC cut-off in the positive direction), and suboptimal individuals (surpassing neither criterion) (Miles et al., 2023).

## 3 Results

### 3.1 Intrusion (cluster B)

**Figure 1:**
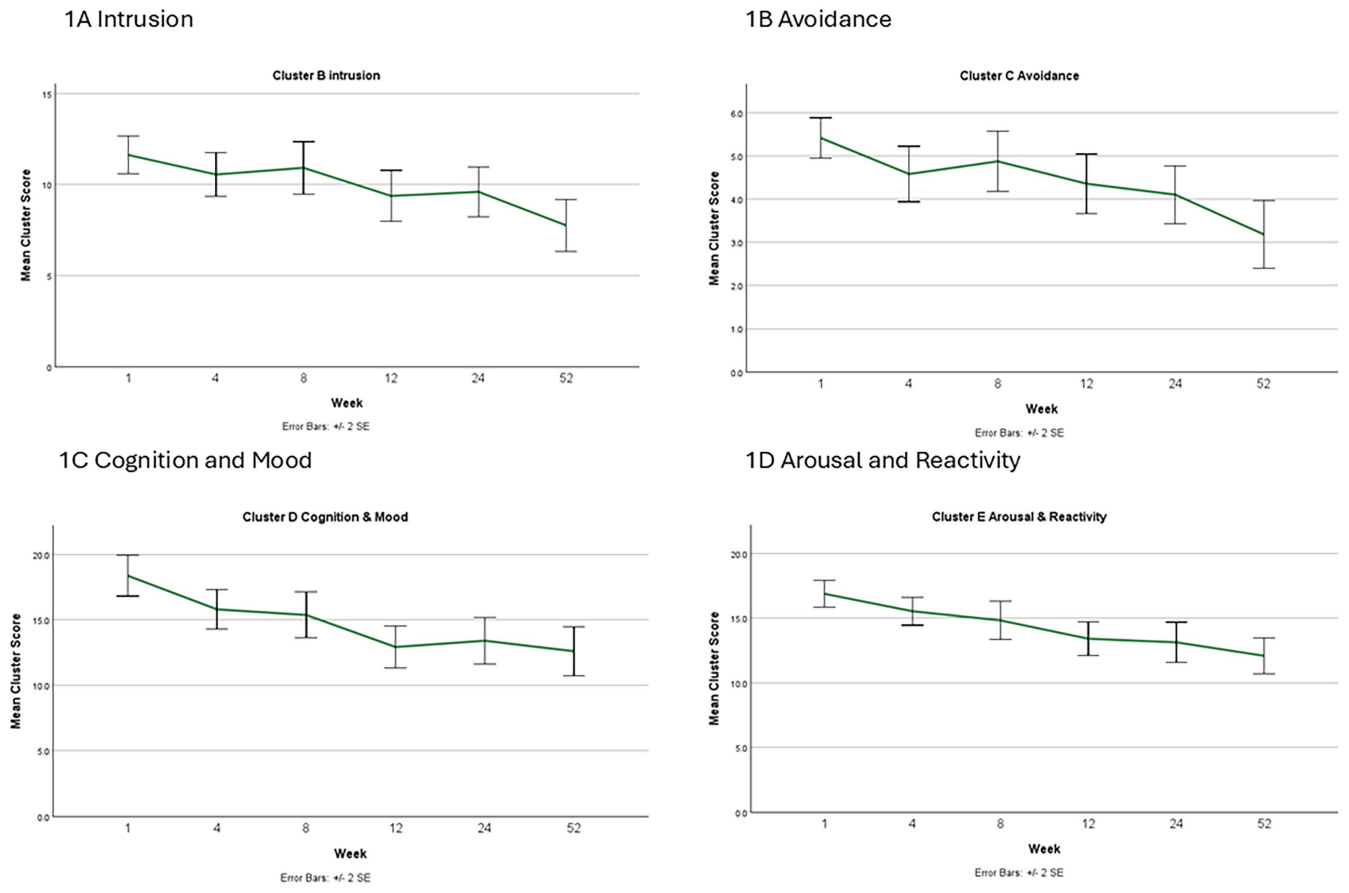
The mean PCL-5 score for intrusion **(A)**, avoidance **(B)**, cognition and mood **(C)**, and arousal and reactivity **(D)** symptoms over time with the standard error (SE) (*N* = 43).

Intrusion scores, analysed using a diagonal covariance structure, decreased significantly over time (*F*(5, 48.98) = 5.29, *p* < 0.001; see Figure 1A for raw values). *Post-hoc* comparisons showed that intrusion scores decreased across most weeks, with a further decrease observed at week 52. This indicates sustained effects after termination of the intervention (Table 2). The number of deployments significantly affected intrusion scores (*F*(1, 30.10) = 4.77, *p* = 0.037), with higher numbers of deployments generally associated with higher intrusion symptom levels. No main effects of group, gender, prior horse experience, or medication use were found, and no interaction effects with week were found. These findings indicate that the decrease in intrusion scores over time was not dependent on the participants’ gender, group, prior experience with horses, existing medication use, or number of deployments.

**Table 2:** Estimated marginal means (EMM) and the standard error (SE) of PCL-5 symptom clusters B, C, D, and E over time points (weeks), with sample size (*N*) per week.

Week	Cluster B		Cluster C		Cluster D		Cluster E	
	EMM	SE	EMM	SE	EMM	SE	EMM	SE
1 (*N* = 43)	11.212	0.728	5.391	0.344	18.334	1.005	16.235	0.726
4 (*N* = 43)	10.166	0.705	4.588	0.407	15.405	1.005	14.572	0.726
8 (*N* = 40)	10.521	0.725	4.806	0.439	15.213	1.008	14.419	0.728
12 (*N* = 42)	8.576	0.780	4.197	0.420	12.751	1.006	12.766	0.726
24 (*N* = 43)	8.806	0.780	3.740	0.385	12.775	1.005	11.683	0.726
52 (*N* = 38)	7.287	0.871	2.909	0.315	12.331	1.007	11.513	0.727

### 3.2 Avoidance (cluster C)

Model fit improved when the interaction effect between week and number of deployments was removed from the final model, with a first-order factor analytic covariance structure. Avoidance scores decreased significantly over time (*F*(5, 66.18) = 15.50, *p* < 0.001, see Figure 1B for raw values). *Post-hoc* comparisons showed that reductions emerged from week 8 and became consistently clear from week 12 onward. In addition, a further decrease at week 52 was observed, demonstrating sustained effects after termination of the program (Table 2). The number of deployments positively affected avoidance scores (*F*(1, 26.78) = 8.43, *p* = 0.007), with higher numbers of deployments generally associated with higher avoidance symptoms. Prior experience with horses also significantly affected avoidance scores (*F*(1, 31.71) = 4.14, *p* = 0.050); participants with prior experience with horses demonstrated higher avoidance symptoms (mean ± standard error: 4.856 ± 0.379) compared with participants without prior horse experience (mean ± standard error: 3.687 ± 0.483). Group membership also significantly affected avoidance scores (*F*(8, 26.35) = 3.01, *p* = 0.015), although differences were inconsistent with large overlapping confidence intervals. No main effects of gender, medication, or interaction effects with week were found.

### 3.3 Cognition and mood (cluster D)

Cognition and mood scores, using a Toeplitz covariance structure, decreased significantly over time [*F*(5, 65.24) = 3.09, *p* = 0.015; see Figure 1C for raw values]. *Post-hoc* comparisons showed that most weeks differed significantly from each other except weeks 4 and 8, and weeks 12, 24, and 52, which did not differ from each other. This indicates that after termination of the intervention at week 12, cognition and mood scores did not decrease further and remained low (see Table 2). The number of deployments positively affected cognition and mood scores [*F*(1,31.53) = 5.62, *p* = 0.024], with higher number of deployments generally associated with higher scores on cognition and mood symptoms. No main effects of group, gender, or medication use, and no significant interaction effects with week were found.

### 3.4 Arousal and reactivity (cluster E)

Arousal and reactivity scores, analysed using AR(1) covariance structure, decreased significantly over time [*F*(5,107.43) = 6.67, *p* < 0.001; see Figure 1D for raw values]. *Post-hoc* comparisons showed that most weeks differed significantly from each other, except week 4 and week 8. After termination of the intervention at week 12, the arousal and reactivity scores did not decrease further but remained low (see Table 2). The number of deployments positively affected arousal and reactivity scores [*F*(1,30.13) = 6.35, *p* = 0.017], with higher numbers of deployments generally associated with higher arousal and reactivity symptoms. Prior experience with horses also significantly affected the arousal and reactivity symptoms [*F*(1,30.17) = 6.57, *p* = 0.016]. Participants with prior experience with horses reported higher symptom levels (mean ± standard error: 14.896 ± 0.698) compared with those without prior experience (mean ± standard error: 12.167 ± 0.888). No main effects of group, gender, or medication use, and no significant interaction effects with week were found.

### 3.5 Reliable change index (RCI) and clinically significant change (CSC)

Of the 43 participants who met criteria for PTSD classification at admission, 30 still met PTSD criteria at 3-month follow-up. Calculating RCI and CSC resulted in a distribution of participants across response categories. In total, 22 participants (51.2%) were classified as suboptimal, as they did not meet the RCI criterion of improving by 12 points or more on PCL-5. In total, 10 participants (23.3%) were classified as improved, with PCL-5 scores decreasing by at least 12 points but not meeting the CSC criterion of <31. Eleven participants were classified as recovered (25.6%), showing ≥12 point improvement and a PCL-5 total score of <31. Table 3 presents the proportion in these three outcome categories that endorsed each of the PCL-5 symptoms as “present” at week 1 and week 24 and the decline in symptoms for the different groups. Percentages reflect the proportion of participants who rated each PCL-5 symptom as ≥2 (“moderate” or higher). The higher proportion of symptoms in the improved group during pre-treatment resulted from the fact that individuals with more symptoms pre-treatment were more likely to be classified in the improved category, even when they showed considerable improvement (Miles et al., 2023).

**Table 3:** The proportion of veterans (*N* = 43) rating each PCL-5 symptom as 2 (moderate) or higher, stratified by treatment outcome group (recovered, improved, and suboptimal).

PTSD symptom	Pretreatment			Posttreatment			Reduction		
	Recovered (*n* = 11)	Improved (*n =* 10)	Suboptimal (*n =* 22)	Recovered (*n =* 11)	Improved (*n =* 10)	Suboptimal (*n =* 22)	Recovered (*n =* 11)	Improved (*n =* 10)	Suboptimal (*n =* 22)
Intrusions	73%	100%	86%	27%	70%	86%	45%pp	**30%pp**	0%pp
Nightmares	45%	90%	82%	36%	70%	95%	9%pp	20%pp	−14%pp
Flashbacks	18%	80%	77%	9%	50%	64%	9%pp	**30%pp**	14%pp
Emotional distress	73%	100%	82%	9%	90%	64%	**64%pp**	10%pp	**18%pp**
Physical reactivity	82%	100%	86%	45%	100%	91%	36%pp	0%pp	−5%pp
Cluster B	58%	94%	83%	25%	76%	80%	33%pp	18%pp	3%pp
Avoid thoughts	73%	90%	86%	18%	80%	86%	55%pp	**10%pp**	0%pp
Avoid activities	91%	100%	100%	27%	90%	82%	**64%pp**	**10%pp**	**18%pp**
Cluster C	82%	95%	93%	23%	85%	84%	**59%pp**	10%pp	**9%pp**
Inability to recall	45%	90%	68%	27%	80%	68%	18%pp	10%pp	0%pp
Negative cognitions	64%	90%	64%	36%	50%	64%	27%pp	**40%pp**	0%pp
Self/other blame	91%	100%	55%	27%	80%	55%	**64%pp**	20%pp	0%pp
Strong negative emotions	82%	100%	73%	18%	70%	77%	**64%pp**	30%pp	−5%pp
Anhedonia	91%	100%	100%	55%	70%	95%	36%pp	30%pp	5%pp
Detachment	91%	100%	95%	55%	60%	91%	36%pp	**40%pp**	5%pp
Numbing	82%	90%	91%	27%	50%	82%	55%pp	**40%pp**	**9%pp**
Cluster D	78%	96%	78%	35%	66%	76%	43%pp	**30%pp**	2%pp
Irritability/aggression	73%	90%	95%	0%	60%	86%	**73%pp**	30%pp	**9%pp**
Impulsivity	18%	70%	50%	9%	30%	55%	9%pp	**40%pp**	−5%pp
Hypervigilance	100%	100%	100%	45%	90%	100%	55%pp	10%pp	0%pp
Startled	82%	100%	95%	18%	80%	95%	64%pp	20%pp	0%pp
Difficulty concentrating	91%	100%	95%	45%	100%	95%	45%pp	0%pp	0%pp
Insomnia	82%	90%	95%	45%	90%	91%	36%pp	0%pp	5%pp
Cluster E	74%	92%	89%	27%	75%	87%	47%pp	17%pp	2%pp

The highlighted rows present averages of the individual symptoms listed above in each cluster, the last column presents week1-week 24 reductions as expressed in percentage points (pp), bold text indicates the largest decrease of a specific question and/or cluster per treatment group.

The recovered group showed a decrease in all symptom clusters, with cluster B (intrusion) showing the smallest reduction, and cluster C (avoidance) showing the largest decrease. The percentage of veterans who maintained symptoms from cluster B with a score of ≥2 decreased from 58 to 25% after the intervention, with symptoms “Flashbacks” and “Nightmares” showing the smallest decrease. For cluster C, a decrease of 59 pp. was found, with both symptoms “Avoiding Thoughts” and “Avoid Activities” decreased by 55 pp. and 64 pp., respectively. Cluster D (negative changes in cognition and mood) showed a decrease of 43 pp., most notably for the symptoms “Strong negative emotions” and “Self/Other blame.” Cluster E (arousal and reactivity) showed a decrease of 47 pp., with “Impulsivity” showing the smallest reduction and appearing less responsive to change.

The improved group showed smaller decreases in symptom clusters compared with the recovered group, with cluster D (negative changes in cognition and mood) being the most responsive to change (30 pp. decrease) and cluster C (avoidance) being the least responsive (10 pp. decrease). The percentage of veterans who endorsed symptoms from cluster B (intrusion) decreased by 18 pp., with the symptom “Physical reactivity” not decreasing at all and the symptoms “Intrusions” and “Flashbacks” showing the greatest decrease. Cluster E (arousal and reactivity) decreased by 17 pp., while “Difficulty concentrating” and “Insomnia” showed no decrease at all.

The suboptimal group showed only minimal decreases, with reduction in cluster B (3 pp.), cluster C (9 pp.), cluster D (2 pp.), and cluster E (2 pp.).

## 4 Discussion

The present study demonstrated that non–trauma-focused EAI was associated with clinically significant decreases across all PTSD symptom clusters in treatment-resistant military veterans. Cluster B and cluster C decreased further after termination of the program at week 12, suggesting sustained effects. Cluster D and cluster E remained stable and did not decrease further after week 12. In addition, the RCI and CSC were calculated, resulting in three treatment outcome groups: suboptimal (51.2%; *n* = 22), improved (23.3%; *n* = 10), and recovered (25.6%; *n* = 11). These findings indicate that 23.3% of the included participants demonstrated statistically reliable but not fully clinically significant improvement, whereas 25.6% achieved both reliable and clinically meaningful symptom reduction. Overall, 51.2% of the participants did not show clinically meaningful improvement.

The findings of this study indicate that all symptom clusters of PTSD decreased significantly over time. In contrast, Malinowski et al. (2018) reported that only cluster E was significantly reduced during EAIs. Studies on trauma-focused treatments (TFT) in non-military populations found improvement across all DSM-5 PTSD symptom clusters (Grasser and Javanbakht, 2019; Melani et al., 2024). However, the literature remains inconsistent regarding the effects of TFT on specific symptom clusters of PTSD. Some studies suggest that TFT is effective in reducing intrusion and avoidance (Schnurr and Lunney, 2015; Zayfert and DeViva, 2004), whereas, others report persistent symptoms across clusters including intrusion (Gross et al., 2024; Levi et al., 2021; Schnurr and Lunney, 2015; Wesseling et al., 2025), avoidance (Wesseling et al., 2025), negative changes in cognition and mood (Wesseling et al., 2025), and arousal and reactivity (Larsen et al., 2019; Miles et al., 2023; Schnurr and Lunney, 2019). These inconsistencies may be explained by differences in study population (such as military vs. non-military), trauma type, measurement methods (such as self-reported vs. clinician-administered, and symptom clusters vs. individual symptoms), and analytic approaches (Wesseling et al., 2025). Although it can be hypothesized that TFT and EAI operate through different mechanisms of change and may differ in which symptom clusters they affect, this study did not provide evidence for this hypothesis. Some preliminary studies suggests that TFT’s may promote recovery from PTSD through different mechanisms of changes (Gallagher and Resick, 2012), such as emotional processing in behavioural (Foa and Kozak, 1986) and restructuring maladaptive cognitions related to trauma in cognitive therapies (Resick et al., 2002), but a systematic review did not confirm these distinctions (Melani et al., 2024). The present study did show differential time effects across PTSD symptom clusters. Scores in cluster B (intrusion) and cluster C (avoidance) continued to decrease after termination of the program, at least up to week 52. Whereas scores in cluster D and cluster E stabilized by week 24 (end of the program). A possible explanation is that EAI reduces avoidance behavior through positive experiential engagement, which may facilitate continued improvements in daily functioning. Perhaps positive experiences during EAI might stimulate veterans to no longer avoid certain stimuli during everyday life. However, the underlying working mechanisms of EAIs remain unclear, and further research is needed to clarify why certain symptom clusters continue to decrease, whereas others stabilize.

At the 3-month follow-up, 13 (30%) of the 43 included veteran participants no longer met diagnostic criteria for PTSD. This rate is comparable to response rates reported in the literature for first-line TFT in military populations (Forbes et al., 2010; Levi et al., 2021; Steenkamp et al., 2015). The RCI and CSC scores resulted in a suboptimal (51.2%, *n* = 22), improved (23.3%, *n* = 10), and recovered (25.6%, *n* = 11) group, which is comparable to studies on the effects of TFT in military populations (Marx et al., 2022; Miles et al., 2023). Thus, 23.3% demonstrated clinically significant improvement, and 25.6% achieved clinically meaningful recovery. However, RCI and CSC thresholds vary across studies (Marx et al., 2022; Miles et al., 2023), supporting the argument of Marx et al. (2022) that these indices should be interpreted as ranges rather than as a single value. Differences in responsiveness across symptom clusters were observed between outcome groups. In the improved group, cluster D was most responsive to change, whereas cluster C was the least responsive to change. In the recovered group, cluster C was the most responsive to change, and cluster B was the least responsive. This is in line with findings in the literature that cluster B (intrusion) is persistent after treatment (Gross et al., 2024; Levi et al., 2021; Schnurr and Lunney, 2015; Wesseling et al., 2025). Future research could further investigate why these differential effects occur for both improved and recovered patients. For now, veterans who benefit from EAIs primarily seem to improve on cluster C and cluster D during the interventions. This pattern is consistent with the non-trauma-focused nature of EAI and may explain the delayed improvements in cluster B, as cluster C and cluster D may exhibit important prerequisites. These findings imply that EAIs could be used as an additive intervention to TFT for treatment-resistant military veterans. The results focused on EAI after first-line treatment when residual symptoms occur. Further research is needed to investigate whether EAI could also be used alongside TFT to enhance tolerance and adherence, for example, by reducing avoidance and cognitive-emotional distress prior to trauma-focused exposure.

A strength of this study is the relatively large sample size and the inclusion of a nine-month follow-up period to measure the long-term effects of the EAI. The most important limitation of this study is that it did not include a control group, which precludes causal inference. Although the longitudinal design allowed participants to serve as their own control, comparisons with standard care or non-treatment conditions were not possible. Confounding variables were minimized by excluding veterans with other ongoing treatments. Additionally, the multi-component nature of the intervention prevents identification of the most effective components. Factors such as social contact with other veterans, interaction with the horse, structured outdoor activities, or evaluation under the guidance of a psychologist during the evaluation at the end of the day may all have contributed. Finally, PTSD symptoms were assessed using the self-report PCL-5 rather than the clinician-administered CAPS-5. Although standard terminology such as ‘recovered’, ‘improved,’ and ‘suboptimal’ was used, residual symptoms across clusters suggest that the term ‘recovered’ may not fully capture clinical reality, consistent with Marx et al. (2022).

## 5 Conclusion

This study demonstrated that EAI was associated with significant improvements across all PTSD symptom clusters in treatment-resistant military veterans. Responsiveness over time differed by cluster. Clusters D (negative changes in cognition and mood) and cluster E (arousal and reactivity) improved primarily during the intervention, whereas cluster B (intrusion) and cluster C (avoidance) showed delayed gains following program completion. In total, 25.6% of treatment-resistant military veterans showed clinically meaningful improvement and had a PCL-5 total score of <31 at week 24 of the intervention. These findings suggest that EAIs may be used as an adjunctive intervention to TFT for military veterans with residual symptoms. Future research should focus on controlled study designs comparing EAIs to waitlist controls and/or other adjunctive treatments. Additionally, evaluating the integration of EAIs alongside first-line treatments and investigating whether such integration can decrease drop-out rates during such treatments would add value.

## Data Availability

The raw data supporting the conclusions of this article will be made available by the authors, without undue reservation.
